# Publishers’ Drawer

**Published:** 1887-04

**Authors:** 


### THE PUBLISHERS' DRAWER.

CREAM OF THE MAILS AND EXCHANGES.

THE WOMAN'S MEDICAL COLLEGE of New York wants $100,000 for a new building.

IT IS RUMORED that Rush Medical College will build a sanitarium on Lookout Mountain for the treatment of consumptives.

THE *St. Louis Medical and Surgical Journal* is authority for the statement that the Post-Graduate School of that city has reduced its fees from $100 to $30.

A NEW BUILDING for the Medical Department of the Western Reserve University of Ohio has been erected for that institution, and presented to it by Mr. John L. Woods, of Cleveland.

THE APPROPRIATION BY CONGRESS of ten thousand dollars toward the conduct of the Ninth International Medical Congress will cover but a small portion of its expenses, but it is thought there will be ample means provided from private sources.

ON AND AFTER APRIL 1, 1887, DR. MAY'S QUIZ-CLASSES, preparatory for the United States Army, Navy and Marine Hospital, General Hospital and Civil Service Medical Examinations, will be conducted conjointly by himself and by Dr. Charles F. Mason, Assistant Surgeon United States Army. This addition has become desirable owing to the great success of the classes, as evidenced by a membership in all classes of over one hundred men during the last three years, with less than five per cent. of failures. The classes will be conducted as heretofore, and the course of instruction will be made still more advantageous by additional facilities, such as, practical instruction and attention to general educational branches. Quizzing by mail. For particulars address, Dr. C. H. May, 202 East 58th St., New York, N. Y.

THE ANNUAL INCREASE OF MEDICAL MEN in this country is over 5½ per cent., while the annual increase of population is less than 2 per cent. Certain statistics of the profession in Illinois show that between the years 1880 and 1886, 7 per cent. of the physicians left the profession and engaged in other pursuits.

JUDGE C. C. FOSTER, of Mecosta county, Michigan, in the case of "State of Michigan v. Vanimmans," decided, when a physician refused to testify on the ground that the evidence would be expert testimony, "After many years' study and observation, I decide that a physician's knowledge is his stock in trade, his capital, and we have no more right to take it without extra compensation than we have to take provisions from a grocery, without pay, to feed the jury. The court rules that the witness is not compelled to testify."

THE OLD ADAGE, says the *Medical Register*, "that 'there is plenty of room at the top,' is cold comfort when we find that in a total of 51,155,783 inhabitants in the United States there are 85,671 physicians. This makes one doctor to every 585 persons, which, as every one who is at all conversant with the facts of the case knows, is entirely too small an average to support a doctor, leaving the family out of the question altogether. In this eighty-five odd thousand physicians there are plenty who have the ability to succeed and make excellent and brilliant practitioners, but who will never get a chance to get near the "top" on account of the crowding, and even if a particularly fortunate one does get a glimpse of this promised land, it is when he is bald, old and gray; it is then too late, and he drops out of life content with only a glimpse of the coveted bourne."

DR. THOMAS LITTLE, OF SPIRIT LAKE, IOWA, in comparing papine with other forms of opium, says: "I have been using papine for the past two months. It meets the requirements of a class in which opiates are indicated, but in which the 'remedy is worse than the disease.' One case in particular has given me a great deal of trouble for years. I have tried opium in every form, and many other narcotics, alone and in combination; but constipation, nausea and nervous prostration have been the invariable results. Some two months since I obtained some papine and commenced on this case with the happiest effect; no nausea, no constipation, no prostration. I have been prescribing it in my practice since with the greatest satisfaction to myself and my patients."

---

THE COST OF INFANT FOODS.

One of the greatest objections that have been made to the use of the various prepared infant foods upon the market has been their high cost. As it will be a matter of interest to the entire profession to know the comparative costs of the various foods, a careful computation of the cost of each class has been made, prepared according to the directions given for infants.

The so-called milk foods or powders are found to be the highest, averaging to cost, when prepared ready for use, about nine cents per pint; next in cost is a class called Liebig's Food, which average six cents or more a pint; next in a class of farinaceous foods, which cost nearly as much as the Liebig Foods. Below all these is Lactated Food, which costs but four cents per pint, making it the most economical food the profession can use. A dollar package of Lactated Food will give an infant one hundred and fifty meals, or sufficient to last about four weeks.

### SUBSTITUTION BY DRUGGISTS.

There was a time when there was no intermediary between the physician and his patient—when every doctor dispensed his own medicines. In cities and closely settled communities this practice gradually became burdensome, and was relegated to a special class, the druggist or pharmacist; but the old-time custom is still adhered to very largely among rural or country physicians.

When the "patent medicine man" made his appearance, this agent or intermediary of the physician promptly assumed the same position towards the intruder, and united to his honorable calling of pharmacist the less honest but probably more profitable one of vender of nostrums. This anomaly would have adjusted itself in time, and, indeed, has already partially done so, but the druggist has been deflected from the straight and narrow path. Aiding a fraud, what more natural than that he should, in certain instances, become imbued with the spirit of fraud? Seeing the gullibility of the public, and knowing the profits accruing from the trade in nostrums, the less honest and more avaricious members of the guild were henceforth but ill-content with the comparatively meager profits of their honorable and legitimate calling. The outgrowth of this spirit was the crying evil of substitution—the replacement of high-priced ingredients in prescriptions by others less costly and totally inefficacious. There is scarcely a physician in our cities and towns who has not, at some time, had good reason to complain of this evil.

Of late the rascally practice has taken a wider range, in a direction made possible by the legitimate advances of the art of pharmacy. We refer to substitution as applied to those products of chemical and pharmaceutical skill, aided by abundant capital, known as "proprietary preparations"—preparations, the nature and ingredients of which are made known to the medical profession, for whose use alone they are manufactured, and which are by no means to be classed or confounded with "patent medicines." Many of these proprietary medicines are of great value commercially, and as a result they are composed of the purest drugs, compounded with great skill. A certain proportion of the medical profession (and some of them men of wide and honorable reputations) have found these preparations good and useful, and their exhibition attended by most satisfactory results; and hence have prescribed them largely, *not the least potent reason for this fact being the feeling of security against substitution induced by the careful and often costly methods of package adopted by the manufacturing chemists.* But as "love laughs at locksmiths" so laughs the substituting druggist at seals and wrappers of unique design, at signatures and brands; and the manufacturing chemist who spends thousands and hundreds of thousands of dollars in keeping up the standard of his preparations, finds himself suddenly accused of allowing them to deteriorate, or possibly of sophisticating them for greater gain.

Without referring to them by name, we may say that very recently a number of the great manufacturing houses have found themselves in this unpleasant position; and in every instance where investigation was possible the fact was disclosed that the apparent deterioration was due to the dishonesty of the retail druggist or prescriptionist who had substituted his own worthless compounds for those ordered by the physician.

Such substitution is not simply dishonest: it is felonious, and displays the same reckless disregard for life that marks the burglar or highwayman who is prepared to take life if it stands in the way of his plunder. The man who does it does not simply filch a few cents from the pocket of his customer (frequently poor and needy), nor does he merely jeopardize the reputation of a physician, but he puts in peril the life of the customer who trusts him.

The honest members of an honorable profession—and fortunately they are largely in the majority—the reputable pharmacists, owe it to themselves to expose these vultures and drive them from the trade. In doing so they should have the aid and countenance of every physician. In the meantime, let every physician not content himself with shunning the shops of those whom he detects in the nefarious habit of substitution, but boldly denounce them, and warn his patients against carrying prescriptions to them. Concerted action of this sort will soon purge the trade of the offending members.—*Editorial, St. Louis Medical and Surgical Journal, March, 1887.*

